# Handheld macroscopic Raman spectroscopy imaging instrument for machine-learning-based molecular tissue margins characterization

**DOI:** 10.1117/1.JBO.26.2.022911

**Published:** 2021-02-12

**Authors:** François Daoust, Tien Nguyen, Patrick Orsini, Jacques Bismuth, Marie-Maude de Denus-Baillargeon, Israel Veilleux, Alexandre Wetter, Philippe Mckoy, Isabelle Dicaire, Maroun Massabki, Kevin Petrecca, Frédéric Leblond

**Affiliations:** a Polytechnique Montreal, Department of Engineering Physics, Montreal, Quebec, Canada; b Centre de recherche du Centre Hospitalier de l’Université de Montréal, Montreal, Quebec, Canada; c Optech, LaSalle, Quebec, Canada; d McGill University, Montreal Neurological Institute-Hospital, Department of Neurology and Neurosurgery, Montreal, Quebec, Canada

**Keywords:** Raman spectroscopy, imaging systems, macroscopic imaging, machine learning, surgical guidance

## Abstract

**Significance:** Raman spectroscopy has been developed for surgical guidance applications interrogating live tissue during tumor resection procedures to detect molecular contrast consistent with cancer pathophysiological changes. To date, the vibrational spectroscopy systems developed for medical applications include single-point measurement probes and intraoperative microscopes. There is a need to develop systems with larger fields of view (FOVs) for rapid intraoperative cancer margin detection during surgery.

**Aim:** We design a handheld macroscopic Raman imaging system for *in vivo* tissue margin characterization and test its performance in a model system.

**Approach:** The system is made of a sterilizable line scanner employing a coherent fiber bundle for relaying excitation light from a 785-nm laser to the tissue. A second coherent fiber bundle is used for hyperspectral detection of the fingerprint Raman signal over an area of $1\;{\mathrm{cm}}^{2}$. Machine learning classifiers were trained and validated on porcine adipose and muscle tissue.

**Results:** Porcine adipose versus muscle margin detection was validated *ex vivo* with an accuracy of 99% over the FOV of $95\;{\mathrm{mm}}^{2}$ in ∼3  min using a support vector machine.

**Conclusions:** This system is the first large FOV Raman imaging system designed to be integrated in the workflow of surgical cancer resection. It will be further improved with the aim of discriminating brain cancer in a clinically acceptable timeframe during glioma surgery.

## 1 Introduction

The current standard treatment for malignant tumors involves the surgical resection of the compromised tissue. Tumor resection can alleviate patient suffering related to tumors pressing on healthy organs^1^^–^^3^ and is shown to improve patient prognosis in breast^4^ and pancreatic^5^ cancer. Improved prognosis is especially true when maximizing the extent of cancer resected since residual cancer cells can lead to tumor recurrence.^6^^–^^9^ Prior to surgery, diagnosis of the tumor is usually achieved through means of palpation, structural imaging [e.g., magnetic resonance imaging (MRI), computer tomography, and ultrasound], molecular imaging (e.g., positron emission tomography), or histology (e.g., biopsy). These methods serve to evaluate the location, severity, and extent of disease, to determine surgical success, and to assess postoperative disease progression. Surgeons then establish their surgical plan and proceed to the tumor site through open or minimally invasive surgery in which the bulk of the tumor is often identified by visual inspection and palpation. Additional tools such as frozen section histology and intraoperative MRI are also used in some tumor sites to further help in assessing disease margins. Tumor grade and location may warrant the resection of complete organs, as can be the case for prostate and breast cancer, while other sites (e.g., brain cancer) require meticulous maximal resection of malignant tissue while preserving the neighboring healthy tissue.^10^ Histopathological analysis of the resected tissue during surgery (i.e., Mohs micrographic surgery) or after surgery is used to assess the presence of positive margins, defined as cancer cells located at the periphery of the resected tissue. It is estimated that 15% to 60% of surgeries for head, neck, liver, oral, breast, and prostate cancers will result in positive margins following the principal surgery.^11^ To improve patient prognosis, positive margins can require an additional surgery from which ensues further patient trauma and complication risks.^12^^–^^14^ On the other hand, it has been suggested that reducing healthy tissue resection can lead to fewer surgical complications, lower the hospital length of stay, and decreased patient discomfort, which motivates tissue conservation surgery in rectal cancer as an alternative to radical surgeries.^15^ It is apparent that extending cancer resection during the principal surgery while preserving healthy tissue is a clinical need for surgical oncology and such a need can be addressed by real-time and *in situ* surgical guidance technology.

Methods showing potential for real-time surgical guidance involve molecular imaging modalities that recover, non-invasively, distinct molecular characteristics of tissue and provide surgeons with tissue-specific information to visualize *in situ* tumor extent. Such methods currently employed for medical treatment include fluorescence-guided surgery (FGS), in which precursors to endogenous fluorescent molecules or fluorescent molecular probes [e.g., 5-aminolevulinic acid (5-ALA) or indocyanine green (ICG)] are administered orally or injected preoperatively into the blood stream. These probes accumulate in organs in different concentrations and emit fluorescence (e.g., green light for ICG) upon illumination with an excitation light source. In glioma surgery, 5-ALA is metabolized preferentially in tumors into the fluorescent protoporphyrin IX to identify residual dense cancer within the surgical cavity. Such approaches have demonstrated their effectiveness by increasing the occurrence of complete resection of the tumor (i.e., no visible contrast in postoperative MRI) in glioma surgery.^16^^,^^17^ However, FGS does not always show surgeons the full extent of cancer in part due to luminescence quenching, fluorescence probe breakdown, or a lack in specificity of probe distribution in healthy and diseased tissue. This translates to absent or hard to detect fluorescence contrast in tissue in which only subtle oncological, morphological, and/or molecular changes occurred.^18^ Studies have shown that postoperative imaging such as MRI, even when employed with contrast agents, is also unable to detect areas associated with a low density of cancer cells associated with tumor infiltrations. When used as a gold standard for evaluating complete tumor resection in glioblastomas, tumor volume measured with MRI may confirm the resection of the tumor, but it does not guarantee the absence of residual cancer cells at the surgical site.^19^^,^^20^ This clinical reality is associated with the high tumor recurrence rate (85%) at the resection margin in glioblastoma patients and its very poor prognostic even when complete tumor resection is achieved.^16^^,^^21^ Efforts are currently being employed to develop new contrast-enhancing molecular probes (e.g., OTL38, LUM015, and SGM-101) with increased specificity for various cancer sites including prostate, breast, and colon.^22^

Label-free molecular imaging (e.g., diffuse reflectance and endogenous fluorescence detection) has gained interest as an alternative surgical guidance solution due in part to its independence from labels and dyes, which require risky, expensive, and lengthy safety studies prior to use in patients. Among label-free molecular imaging technologies, Raman spectroscopy (RS) stands out with its ability to recover a molecular specific fingerprint from interrogated tissue. RS is capable of discriminating in real-time cancer from normal tissue^23^ and has been shown to be superior to diffuse reflectance and endogenous fluorescence combined in discriminating between breast tissue histopathologic categories.^24^ This non-invasive and non-destructive method employs a laser source to interrogate the intrinsic molecular vibrations of tissue content, i.e., DNA, lipids, and proteins, and exploits this tissue-specific information to map tissue heterogeneity accordingly. RS relies on recovering signal from inelastic scattering in interrogated samples, a relatively rare event (∼1 in $10^{7}\text{ photonic}$ events) that is often difficult to decouple from high-intrinsic tissue fluorescence. Improvements in data preprocessing methods, spectrometer resolution, camera sensitivity, and laser excitation sources have led to the development and implementation of RS systems for *in vivo* discrimination of tissue.^25^ RS for surgical guidance has been demonstrated with handheld single-point probes in humans and has already shown across several studies its potential to extend cancer resection effectively following the main tumor resection such as during glioma surgery,^25^ gastrointestinal inspections,^26^ early lesions detection in oral cancer,^27^ and breast cancer lumpectomy.^28^ However, point measurements do not offer spatial context, which can make difficult the identification of margins and localized cancer infiltrations linked to subtle molecular changes.^29^ The need for spatial context motivates the development of Raman imaging to accomplish the task of identifying residual cancer and its margins. Surgical time constraints and user adoption impose the challenging requirement of making Raman imaging a rapid modality that can quickly interrogate large surgical cavities (i.e., several ${\mathrm{cm}}^{2}$).

Many Raman microscopes have been developed with the intention of guiding surgery by assisting pathologists in the visualization of tissue margins in excised tissue (e.g., breast,^30^ skin,^31^ and oral^32^ cancer). These systems can cover areas up to several ${\mathrm{mm}}^{2}$, but they are unsuitable for performing molecular imaging *in vivo*. Handheld stimulated Raman scattering microscopy systems have been developed and tested successfully to address precise tissue margin localization *in vivo* in mice for skin^33^ and brain^34^ cancer. These systems have a restricted field of view (FOV), i.e., $<4\;{\mathrm{mm}}^{2}$, which requires numerous measurements to cover the entire surgical area.^35^ It was demonstrated by our group in 2016 that coherent fiber bundles, which coherently relay images from one end to the other, can be used for Raman imaging probes for the purpose of wide-field and line scanning collection in biological tissue.^36^ The most recent system had an FOV of $14\;{\mathrm{mm}}^{2}$ and spectral resolution of $6\;{\mathrm{cm}}^{-1}$. It was shown that acquisition times of 700 to 900 ms per line were required to discriminate, based on tissue Raman band ratios, porcine adipose from muscle tissue, as well as bovine white and gray matter.^37^ Raman imaging over a large FOV was also validated *in vivo* in mice with the use of injected nanoparticles for signal enhancement.^38^ However, there remains a lack of Raman imaging systems with large FOV for *in vivo* human clinical use. The only Raman imaging system with a large measurement area ($1\;{\mathrm{cm}}^{2}$) that was developed and tested for a human *in vivo* application is reported by Schmälzlin et al. (2018)^35^ for skin cancer although no cancer margin detection was demonstrated.

Here we present the design and characterization of a novel Raman line scanning system developed with the aim of being integrated in a human clinical study. The line scanning handheld imaging probe has a spatial resolution of 250  μm and a spectral resolution of $6\;{\mathrm{cm}}^{-1}$ allowing Raman imaging to be performed over an FOV of $95\;{\mathrm{mm}}^{2}$. The probe is sterilizable and acquires spectra in the fingerprint region from 400 to $1900\;{\mathrm{cm}}^{-1}$. It is designed to accommodate an apparatus for contact measurement with a head diameter of 20 mm while offering non-contact measurements with a working distance of 40 mm. A bright-field channel allows for visualization of tissue structures under white-light reflectance. The probe also uses a flexible coherent fiber bundle for collection and is the first to use a flexible fiber bundle for custom laser profile excitation, e.g., a scanned laser line. The system optical design and corresponding performance metrics, i.e., laser line profile and optical transfer functions (OTFs), employed for design validation are first presented. Raman hyperspectral images of porcine adipose and muscle tissue were acquired, and band assignments are presented with their corresponding biological interpretation in terms of molecular bonds. The system’s ability to discriminate between tissue types is then demonstrated using support vector machine (SVM) technology. A detailed photonic budget analysis is finally presented comparing the system against a proven handheld single-point surgical guidance Raman system.^39^ The future development steps required to achieve *in vivo* human tissue imaging are finally discussed to obtain a comparable level of Raman signal-to-noise ratio (SNR) within an acceptable clinical timeframe.

## 2 Methods

### 2.1 Imaging System

**Fig. 1:**
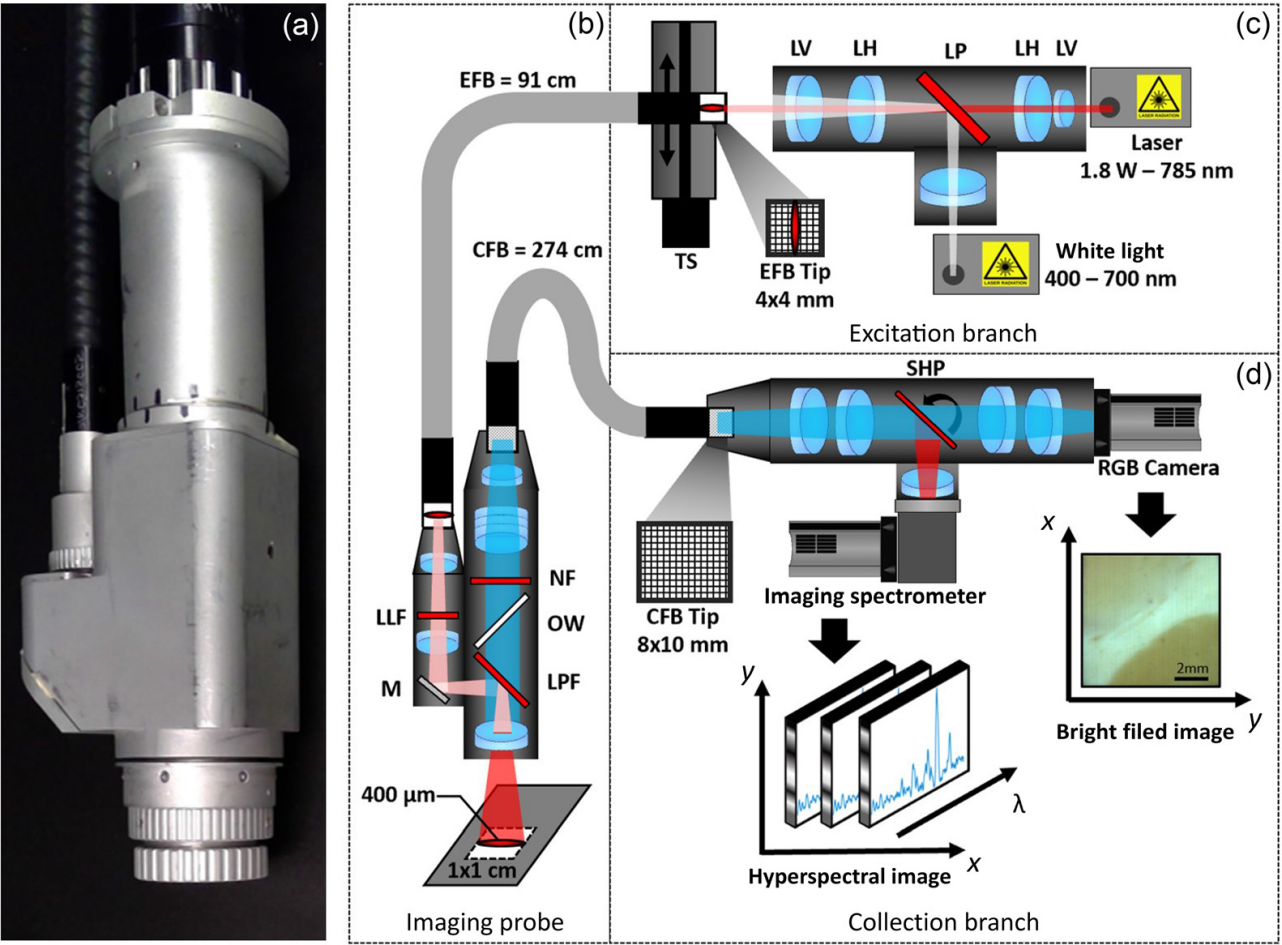
(a) Handheld Raman imaging probe and schematic of the imaging system consisting of (b) the imaging probe with its EFB, a series of optics [LLF, mirror (M), and long-pass filter (LPF)] to propagate white light and laser light onto the sample to interrogate the tissue, a series of optics (LPF, optical window, and notch filter) to collect the generated Raman signal and reflected white light, and a CFB; (c) the excitation branch generating a line-shaped laser excitation at the entrance of the EFB mounted on a TS while also shining white light into the EFB; and (d) the collection branch capturing white light and Raman signal from the CFB, separating the two optical components with a scanning high-pass filter, obtaining a white light image on an RGB camera while extracting spectral features with an imaging spectrometer. All illustrated lenses are spherical unless labeled LH for cylindrical lenses with their curvature in the horizontal direction or LV for cylindrical lenses with their curvature in the vertical direction.

The line scanning system consists of three main parts (Fig. 1): an excitation branch, an imaging probe, and a collection branch. Each component of the system is controlled via custom software developed using LabVIEW (National Instruments, USA) and a graphical user interface. Components including the laser, the broadband source, a motorized translation stage (TS), a galvanometer, a charge-coupled device (CCD) camera, and a color (RGB) camera are synchronized with the help of a digital acquisition device (USB-6351, National Instruments, USA).

The excitation branch [Fig. 1(c)] consists of light sources for RS or bright-field imaging, a series of optics, and a motorized linear TS (A-LSQ075A-E01, Zaber, Canada). The excitation branch generates either a scanning laser line or a widefield white light illumination injected in a $4\times 4\;{\mathrm{mm}}^{2}$ imaging fiber bundle (IG-154, Schott, USA). The laser source is a custom 785 nm continuous-wave laser (Innovative Photonic Solutions, USA) with 1.8-W power, a spectral width of <0.2  nm, and a numerical aperture (NA) of 0.5. The broadband source consists of a 3000-K light emitting diode (LED) (MWWHL4, Thorlabs, USA) that is turned off during Raman measurements to avoid contamination. Optics (cylindrical lenses and dichroic mirror) are used to generate a laser line on the surface of an imaging fiber bundle, with the secondary role of coupling the 785-nm laser line and widefield white light sources into the fiber bundle. Finally, the imaging bundle is mounted onto a motorized TS responsible for repositioning the bundle surface relative to the laser line and thus ensuring line scanning in the excitation branch.

The imaging probe [Fig. 1(b)] consists of a series of illumination optics and imaging optics. Illumination optics include an excitation fiber bundle (EFB) to reproduce the laser excitation pattern from the excitation branch end of the excitation bundle to the probe end and a laser line filter (LLF) centered at 785 nm (LL01-785-12.5, Semrock, USA) with a transmission spectral range from 425 to 600 nm. The excitation pattern (laser line or widefield) is then projected through the probe illumination optics onto the sample at a working distance of 40 mm. The imaging optics include a series of optics designed to collect white light (400 to 650 nm) as well as fluorescence and Raman scattering (810 to 922 nm) through another fiber bundle. A dichroic mirror allows for a maximum reflection of the laser source onto the sample while maximizing fluorescence/Raman collection efficacy. The dichroic mirror ensures that a portion of broadband light (50%) is reflected onto the sample and collected for bright-field imaging. The probe was designed to accommodate a sterilizable and detachable nose with a Raman grade ${\mathrm{MgF}}_{2}$ window (i.e., with no Raman peaks between 400 and $1900\;{\mathrm{cm}}^{-1}$ and low intrinsic fluorescence) (Batch #61973, Crystran, USA). This was designed for future *in vivo* applications for which ensuring tissue contact might be more practical. In the overall optical design, mobile parts, electronics, and mechanical components were avoided to facilitate cleaning and sterilization and to ensure patient safety. The probe [Fig. 1(a)] weighs 0.6 kg without the fiber bundles and has a volume of ∼4.5 (width) × 7 (height) × 18 (length) cm.

The collection branch [Fig. 1(d)] is composed of a collection fiber bundle (IG-163, Schott, USA), a series of optics, a dichroic mirror (FLD 748 DSP, Iridian, Canada) mounted on a galvanometer scanner (6870M, Novanta, USA), a spectrometer (HT, EmVision, USA), a cooled CCD camera (Newton 920, Oxford Instruments, USA), and a high-sensitivity CMOS RGB camera (DCC1240C, Thorlabs, USA). The CFB transmits the bright-field image and fluorescence/Raman signal collected by the imaging probe to the collection branch. From the bundle collection end, the optics relay the collected image onto the dichroic mirror galvanometer through which visible light is transmitted through a series of optics into the RGB camera for bright-field imaging. Raman scattering (810 to 922 nm or 400 to $1900\;{\mathrm{cm}}^{-1}$) is reflected into the Raman spectrometer, and spectral components are separated onto the cooled (−70°C) camera sensor. The image captured by the sensor thus possesses a spatial axis (y axis) and spectral axis (λ axis). The rotation of the mounted dichroic mirror controls which area of the imaging probe’s FOV (x axis) is aligned with the spectrometer slit and is synchronized with the excitation branch’s TS position during measurements.

### 2.2 Optical Design

Specific lens sizes and focal lengths were determined via the optical design software Zemax (Zemax, USA) to meet requirements in each of the system subcomponents (i.e., excitation branch, imaging probe, and collection branch). Off-the-shelf components were selected when applicable.

The excitation branch requirements involved producing a uniform 100  μm (x axis) × 4 mm (y axis) laser excitation profile at the entrance of a 4×4  mm excitation bundle, from a multimode fiber of 400  μm core diameter with 0.22 NA to be relayed through the $4\times 4\;{\mathrm{mm}}^{2}$ EFB into the imaging probe. The laser line profile relative intensity was first simulated with the optical design software and was then measured at the face of the EFB with an RGB camera.

The imaging probe optical design was subdivided into two parts: the illumination branch and the imaging branch, although they had some optical components in common. Lenses and their positions within the illumination and imaging branches were selected according to the resulting OTF and evaluated through the optics of each branch, from sample to bundle face, across the circumradius of each branch’s fiber bundle face ($4\times 4\;{\mathrm{mm}}^{2}$ for the illumination branch and $10\times 10\;{\mathrm{mm}}^{2}$ for the imaging branch). The normalized OTF was evaluated using the optical design software and used to optimize the spatial resolutions (along the x and y directions) of the system. The optimal optical configurations were those for which the OTF—for specific spatial resolutions and wavelengths—were above 0.5 across the entire circumradius of the fiber bundles. The illumination branch’s targeted spatial resolution for 785 nm was set at 2  cycles/mm or 250  μm. No resolution limit was set for visible light (400 to 650 nm) illumination since the excitation branch would generate widefield broadband illumination of the sample. The imaging branch’s target spatial resolution for bright-field imaging (400 to 650 nm) was set at 10  cycles/mm or 50  μm while the minimum resolution for Raman fingerprint imaging (800 to 940 nm) was set at 2  cycles/mm or 250  μm. An additional design criterion for the imaging branch consisted of maintaining a >70% relative illumination profile across the circumradius of the FOV of the imaging probe.

The collection branch requirements included maintaining the bright-field image spatial resolution relayed by the CFB of at least 10  cycles/mm or 50  μm onto the sensor of the RGB camera while also maintaining the relayed Raman image’s spatial resolution of 2  cycles/mm or 250  μm onto the 75  μm (x axis) × 7.2 mm (y axis) slit of the Raman spectrometer. The selected optics were deemed acceptable if the OTF for the above-mentioned spatial resolutions and their corresponding wavelengths was above 0.5 for the entire circumradius of the white light.

### 2.3 Raman Imaging of Biological Samples

The biological sample used for this study was a pork chop from a butcher shop, 1-cm-thick, flat, and with a visually clear margin between adipose and muscle tissue at its surface. The tissue was placed over a low Raman activity aluminum slide (Miro5011, Anomet, Canada) to prevent any background generated from under the sample. Raman images were acquired in three separate areas of the sample: muscle, adipose, and a margin essentially composed of a mixture of adipose and muscle tissue. Bright-field images of the sample were visually inspected, and all pixels were labeled as either pure adipose, pure muscle, or tissue margin. The sample was humidified with buffered blood bank saline (312-651, Thermo Scientific, USA) drops between measurements. Bright-field images before and after line scanning were acquired to confirm that the sample margins had not shifted during measurements and the sample’s color had not changed, which would have been indicative of tissue damage.

The measurement parameters are presented in Table 1. The entire FOV of the system ($95\;{\mathrm{mm}}^{2}$) was imaged by scanning a total of 40 lines on the sample. Binning over 6 pixels along the spatial axis of the CCD (y axis) was done to increase SNR, resulting in a 250-μm effective spatial resolution. Translation distance between line measurements was 250  μm. Binning along the spectral axis (3 pixels) also increased SNR with minor degradation of the spectral resolution. The resulting hyperspectral cube dimensions from line scanning were 40 (x axis) × 42 (y axis) × 341 (λ axis). The laser line profile was of a 400  μm×9.5  mm with a total power of 230 mW corresponding to an average intensity of $6.1\text{ W}/{\text{c}m}^{2}$ at the sample surface. The luminescence (i.e., Raman and background fluorescence resulting from 785-nm laser line illumination) from 400 to $1900\;{\mathrm{cm}}^{-1}$ (810 to 922 nm) was collected for each line during a 5-s exposure time. An external broadband light LED was used to uniformly illuminate the sample. The bright-field images were used as a reference to place the sample at the focus of the imaging system. The broadband source, along with any other sources of ambient light, was turned off throughout the duration of the line scanning measurements.

**Table 1:** Raman imaging system technical parameters selected for the *ex vivo* experiments.

	Specifications
FOV	$95\;{\mathrm{mm}}^{2}$
Working distance	40 mm
Number of lines	40 lines (9.5 mm×400 μm each)
Translation distance	250 μm between line measurements
Spatial resolution NIR	x axis: 250 μm and y axis: 250 μm
Spectral resolution NIR	$6\;{\mathrm{cm}}^{-1}$ (at $1085\;{\mathrm{cm}}^{-1}$)
Number of pixels	x axis: 40, y axis: 42
Spectral range	λ axis: 400 to $1900\;{\mathrm{cm}}^{-1}$
Exposure time	Adjustable but 5 s per line (total: 200 s) in this study
Spatial resolution bright field	50 μm

### 2.4 Spectral Data Preprocessing

The data preprocessing pipeline began with applying a median filter to each spectrum of the image to eliminate cosmic rays. Following this, a Raman shift (λ axis) calibration was performed from a reference measurement of acetaminophen, which has a very well characterized Raman spectrum with low background and is one of the Raman shift standards identified in the American Society for Testing and Materials’s (ASTM) Standard Guide for Raman Shift Standards for Spectrometer Calibration (ASTM E1840). This calibration was achieved by performing a cubic spline interpolation using the six most prominent peaks from the acetaminophen Raman fingerprint spectrum. The spectra were then corrected for system response and relative intensity variation across the image with a smoothed reference measurement on a NIST standard for 785-nm excitation (SRM 2241, NIST, USA). The NIST reference measurement was smoothed using a 2D Gaussian filter of second order to reduce artifacts introduced by scratches on the NIST standard. Spectra at the borders of the corrected hyperspectral cube were rejected due to border artifacts generated by the Gaussian filtered system response. A fifth-order polynomial fit was then applied on each system-corrected spectrum to approximate the background before removing it with a rolling-ball algorithm^40^ using a ball size of 16. Spectra were then normalized using the standard normal variate method prior to use for training, validating, and testing a classifier to distinguish adipose from muscle tissue.

### 2.5 Classification

As a proof of concept for tissue margin detection, two classification algorithms were used: SVM and random forest (RF). The classifiers were trained and validated to perform pixel-wise classification (i.e., no spatial features were used, only spectral ones). The training set consisted of half an image of adipose tissue (20 lines) and half an image of muscle tissue (20 lines). The validation set was composed of the remaining half of the image of adipose tissue (20 lines), the remaining half of the image of muscle tissue (20 lines), and the entire image of a tissue margin (40 lines). The Raman images of only adipose or muscle tissue consist of an average of 10 acquisitions (total acquisition time of 2000 s) at the same spatial location to maximize SNR. The margin image [Fig. 6(a)] consists of a single Raman image acquisition (total acquisition time of 200 s) in which the margin location is centered in the system FOV to ensure an even class distribution. Assigning class labels to images of solely porcine adipose or muscle tissue was straightforward since each image contained one class, whereas labeling of the tissue margin was executed visually using tissue color and structure changes across the bright-field image. In total, the training set consists of 1482 spectra (741 for each class) and the validation set consists of 2964 spectra [741 for each class and 845 (adipose) + 637 (muscle) from the margin image]. Including data from adipose, muscle, and margin images into the validation set forces the models to learn to classify spectra coming from single-class images (i.e., only adipose or muscle tissue) and from margin images for which there might be interclass cross-talk.

The classification models were built by constructing the following pipeline: feature selection, feature standardization, and classifier development. The feature selection step allows for reducing the dimensionality of the dataset to simplify and improve the classifier performance. Here a linear SVM penalized with the L1-norm regularization (L1-SVM)^41^ was used as a feature selector. L1-SVMs have sparse solutions: many weights assigned to the features are zero. Features having non-zero weights were kept for model building. Then features were independently standardized by subtracting their mean and dividing by their standard deviation. The final step of the pipeline is the classification model (SVM or RF). SVMs with two different kernels were explored: the linear and the radial basis function (RBF) kernels, where the optimized hyperparameters consisted of the regularization parameter (C) with the values C = [0.001, 0.01, 0.1, 1, 10] evaluated for both kernels and the gamma parameter (γ) with the values γ = [0.001, 0.01, 0.1, 1, 10] evaluated for the RBF kernel. The RF optimized hyperparameters that were evaluated were the number of trees (n_estimators) using the values n_estimators = [100, 200, 300] and the subsample size (max_samples) using the values max_samples = [0.6, 0.7, 0.8]. A grid search was performed to obtain the optimal hyperparameters for both models. These hyperparameters consisted of parameters given to models to control the learning process (i.e., to avoid under/overfitting). During the grid search, for a given set of hyperparameters, the model was trained on the training data and then validated on the validation set. Optimal hyperparameters were chosen based on the validation accuracy of both models. The machine learning algorithms employed for this classifier-generating pipeline were retrieved from the Python library Scikit-learn.^42^ The model was trained and validated with training and validation sets independent of each other.

The model having the optimal validation accuracy was kept, and its pixel-wise predictions on the margin image along with the corresponding class probabilities of each pixel were computed to visually interpret the classification results. Class probabilistic outputs were computed using Platt scaling,^43^ resulting in probability scores varying from 0.5 to 1.0, where 1.0 corresponds to an absolute class membership probability and 0.5 is the lowest class membership probability. The algorithms used for class probability assignment were retrieved the Python library Scikit-learn.^42^ No testing set was available in this study.

## 3 Results

### 3.1 Optical Design

**Fig. 2:**
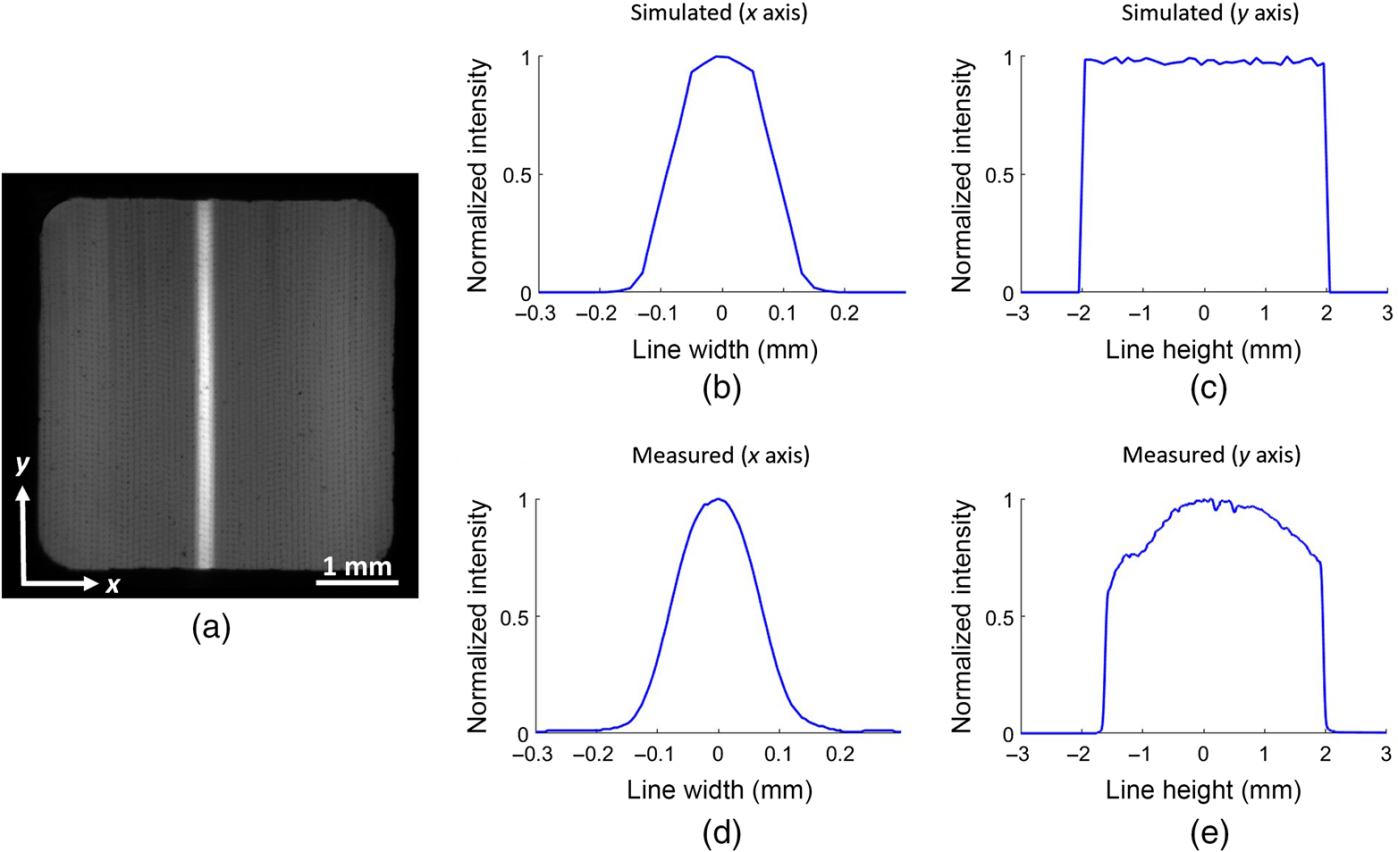
(a) Laser line profile relayed through the EFB and imaged at the distal face of the EFB. Simulated laser line profile generated by the excitation branch at the distal end of the 4×4  mm fiber bundle on the (b) x axis and (c) y axis. Measured laser line profile on the (d) x axis and (e) y axis.

The optical design of each subcomponent of the imaging system, the excitation branch, the imaging probe, and the collection probe, was evaluated individually. Figure 2 presents the laser line profile generated by the excitation branch through the EFB. The laser line profile was simulated according to the optical configuration within the excitation branch, showing the theoretical excitation laser line profile at the distal end of the fiber bundle with an x axis (line width) of 160  μm and a y axis (line height) of 4 mm. The measured normalized intensity profile was obtained for the x axis and the y axis, resulting in a laser line width of 160  μm and line height of 3.8 mm. Line width and line height are characterized according to their measured full-width at half-maximum. There is a slight disparity of intensity distribution at the extremities of the laser line height between the real and simulated laser profiles.

**Fig. 3:**
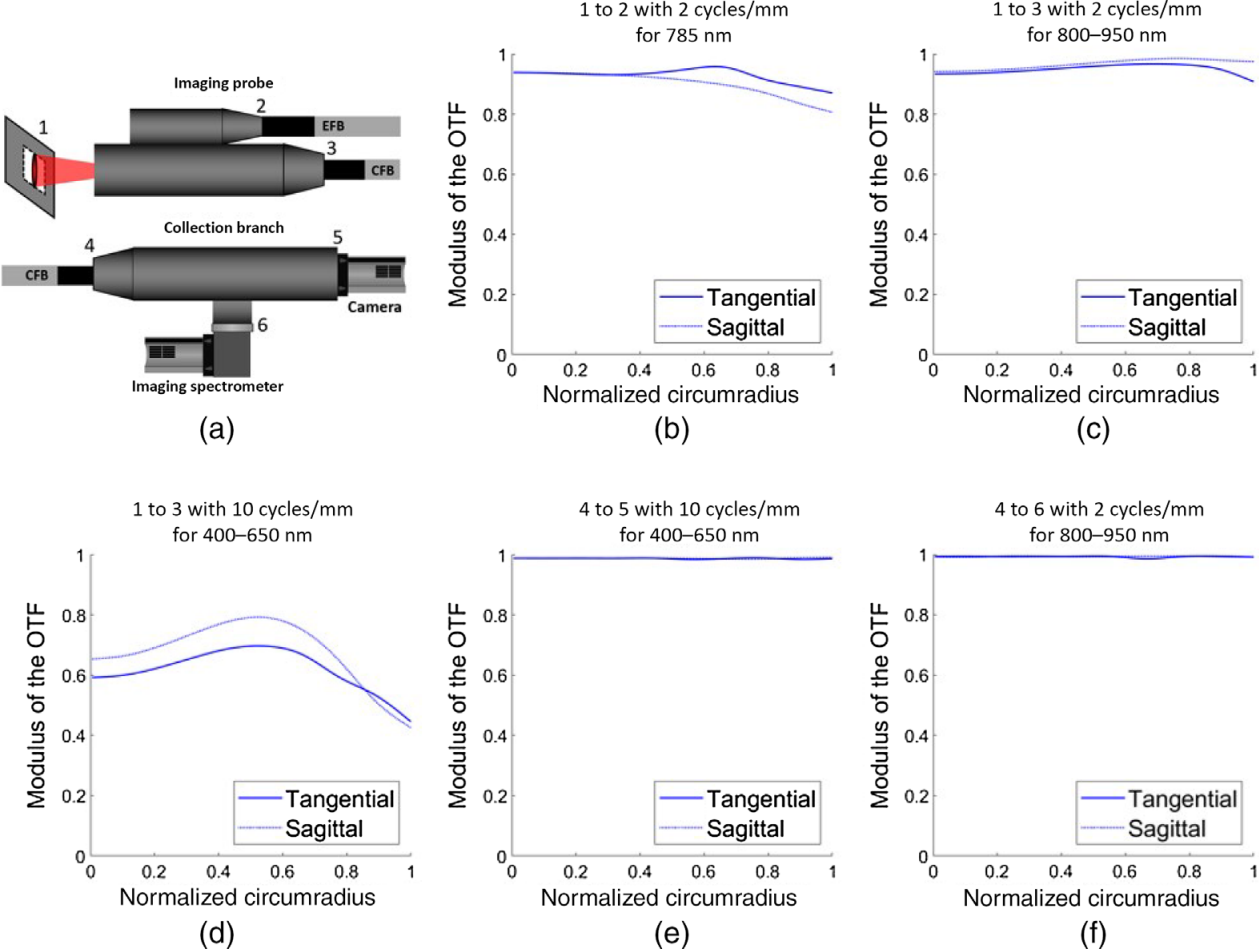
(a) Schematic of the imaging probe and collection branch illustrating components marking the start and end of the optical path selected for OTF simulation. (b) OTF of the imaging probe from 1 to 2 for a spatial resolution of 2  cycles/mm and for an illumination wavelength of 785 nm. OTF from 1 to 3 for (c) IR light (800 to 950 nm) at a spatial resolution of 2  cycles/mm and (d) visible light (400 to 650 nm) at a spatial resolution of 10  cycles/mm. (e) OTF from 4 to 5 for visible light (400 to 650 nm) at a spatial resolution of 10  cycles/mm (black) and (f) from 4 to 6 for IR light (800 to 950 nm) at a spatial resolution of 2  cycles/mm.

The OTF was evaluated for five optical configurations [Fig. 3(a)]. Figure 3(b) presents the OTF of the imaging probe from the imaging plane to the EFB face across its circumradius for a spatial resolution of 2  cycles/mm (or 250  μm) and an illumination wavelength of 785 nm. The OTF of the imaging probe from the imaging plane to the CFB face across its circumradius is shown for Fig. 3(c) IR light (800 to 950 nm) at a spatial resolution of 2  cycles/mm (or 250  μm) and for Fig. 3(d) visible light (400 to 650 nm) at a spatial resolution of 10  cycles/mm (or 50  μm). Figure 3(e) presents the OTF of the collection branch from the collection bundle face to the RGB camera for visible light (400 to 650 nm) at a spatial resolution of 10  cycles/mm (or 50  μm) (black). Figure 3(f) shows the OTF from the collection bundle face to the spectrometer slit for IR light (800 to 950 nm) at a spatial resolution of 2  cycles/mm (or 250  μm). Tangential and sagittal polarization of light are presented for each OTF simulated. OTF modulus remained above 0.5 in most optical configurations for their respective target spatial resolutions.

### 3.2 Raman Imaging of Biological Samples

**Fig. 4:**
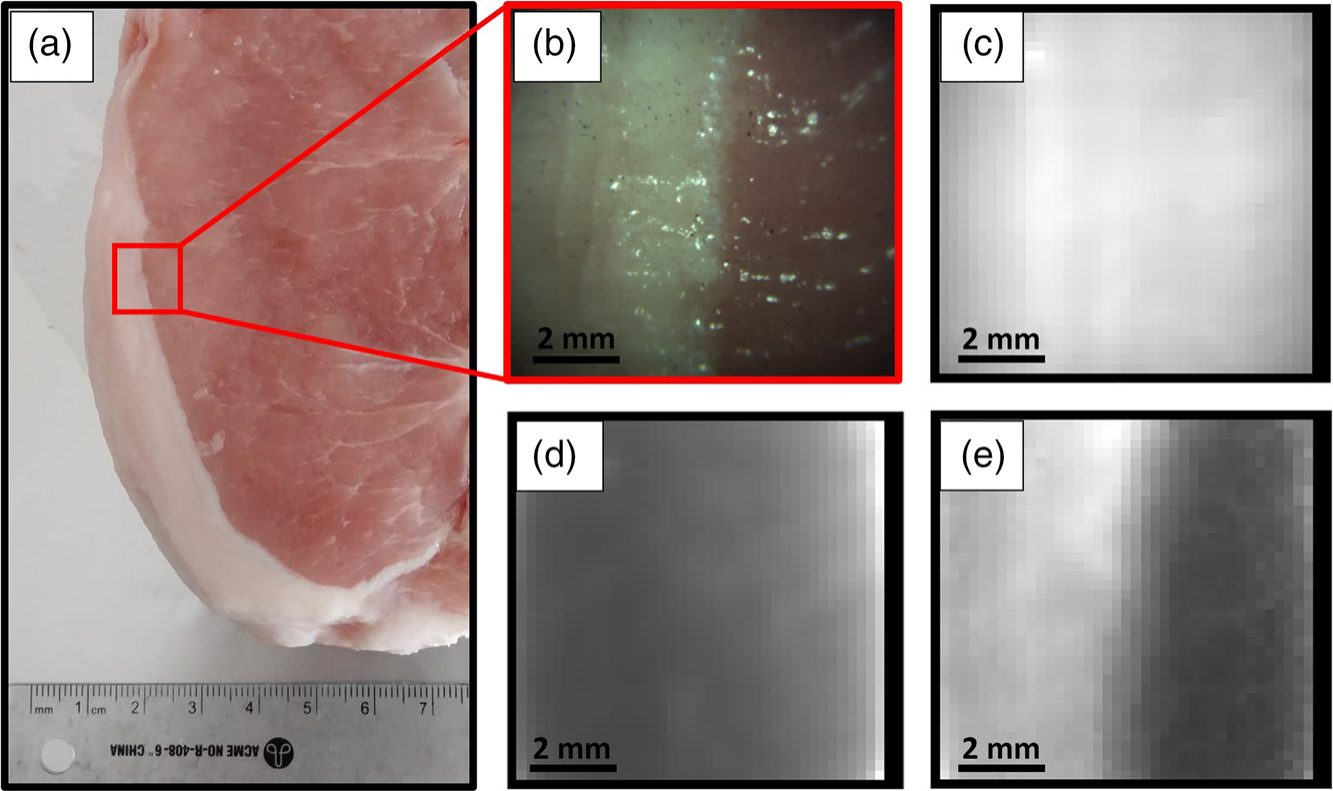
Study of the imaging system using a pork chop: (a) photograph of the full pork chop; (b) bright-field image of the FOV; (c) uncorrected line scan luminescence (Raman and background fluorescence resulting from 785 nm laser line illumination) intensity image; (d) line scan background intensity image; and (e) line scan Raman intensity image. Each intensity value within the intensity images corresponds to the integral over the entire spectral range of the system. Black column on the right of (b)–(d) corresponds to pixels with relative illumination intensity of <40%.

Figure 4 shows a photograph of the porcine sample interrogated with the imaging system: a bright-field image, an uncorrected (no calibration or preprocessing applied) line scan luminescence (Raman and background fluorescence resulting from 785-nm laser line illumination) intensity image, an estimated line scan background intensity image, and a line scan Raman intensity image. Each intensity value within the intensity images corresponds to the integral over the entire spectral range of the system. Although no tissue contrast is perceived in the overall luminescence and the background intensity images, there is a visually distinct contrast of Raman scattering intensity between porcine adipose and muscle tissue.

**Fig. 5:**
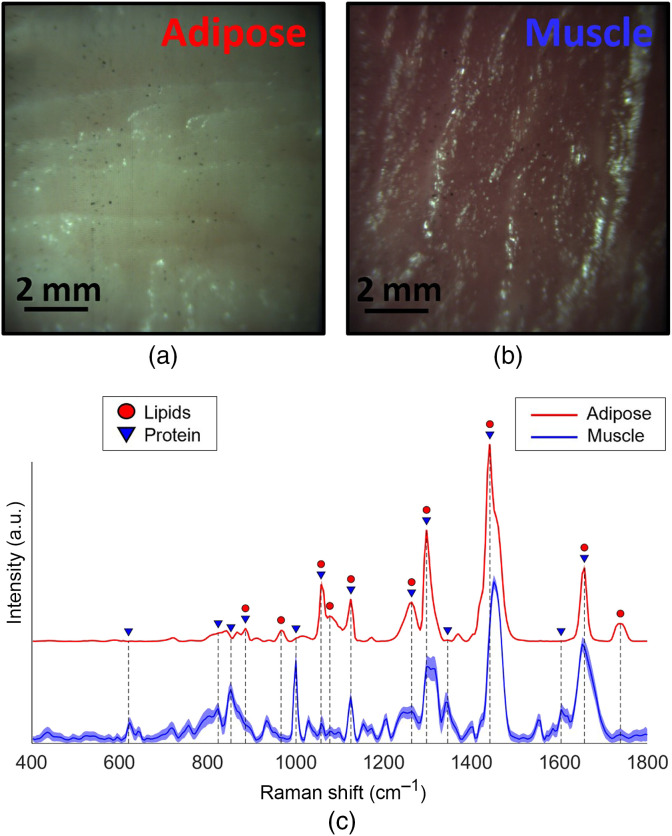
Training set imaged samples: (a) bright-field image of porcine adipose tissue; (b) bright-field image of porcine muscle; and (c) corresponding averaged spectrum and standard deviation (shading) of each tissue obtained following 10 repeat measurements. Blue triangles designate Raman bands associated with protein content while red circles designate Raman bands mostly associated with lipid content. An arbitrary vertical offset was added to the adipose tissue mean spectrum and standard deviation to facilitate the visualization of the spectra from both tissue. Variations across adipose tissue spectra are too small to observe the standard deviation (red shading) in the figure.

Figure 5 presents the average Raman spectra and standard deviation of the training data set for this study showing common and distinct spectral features (i.e., Raman bands) associated with protein and lipid content for each tissue type. Several spectral features and their biological relevance are shown in Table 2. From these spectra, several classifiers were trained. and the spectral features most important for tissue discrimination were extracted from the L1-SVM feature selector.

**Table 2:** Prominent Raman features for porcine muscle and adipose tissue. Bands highlighted in boldface correspond to peaks most relevant to tissue classification.

Raman band (${\mathrm{cm}}^{-1}$)	Molecular bonds	Molecular family	Reference
620	C─C twist of phenylalanine	Protein	44 and 45
824	C─C stretch of proline and hydroxyproline	Protein	44 and 45
853	C─C stretch of proline and tyrosine ring	Protein	44 and 45
886	${\mathrm{CH}}_{2}$ rocking	Lipid and protein	44 and 45
967	${\mathrm{CH}}_{2}$ out of plane bend and C─C wagging	Lipids	44 and 46
**1001**	**Phenylalanine breathing mode**	**Protein**	44 and 45
1063	C─C anti-symmetric stretch and C─N Stretch	Lipid and protein	44 and 46
1082	C─C stretch and C─N stretch	Lipid	44 and 46
1126	C─C symmetric stretch and C─N stretch	Lipid and protein	44–46
1264	$═{\mathrm{CH}}_{2}$ in plane deformation and amide III band	Lipid and protein	44–46
1298	${\mathrm{CH}}_{2}$ twist	Lipid and protein	44–46
1346	CH deformation and ${\mathrm{CH}}_{2}/{\mathrm{CH}}_{3}$ wagging	Protein	44 and 45
**1442**	${\mathrm{CH}}_{2}$**symmetric deformation**, ${\mathrm{CH}}_{2}/{\mathrm{CH}}_{3}$**antisymmetric deformation, and**${\mathrm{CH}}_{2}$**Bending**	**Lipid and protein**	44–46
1604	C═O stretch of amide I and C═C bending mode in tyrosine and phenylalanine	Protein	44 and 45
1657	Amid I band, C═C stretch and C═O stretch	Lipid and protein	44–46
1739	C═O ester stretch	Lipid	44 and 46

### 3.3 Margin Identification

**Fig. 6:**
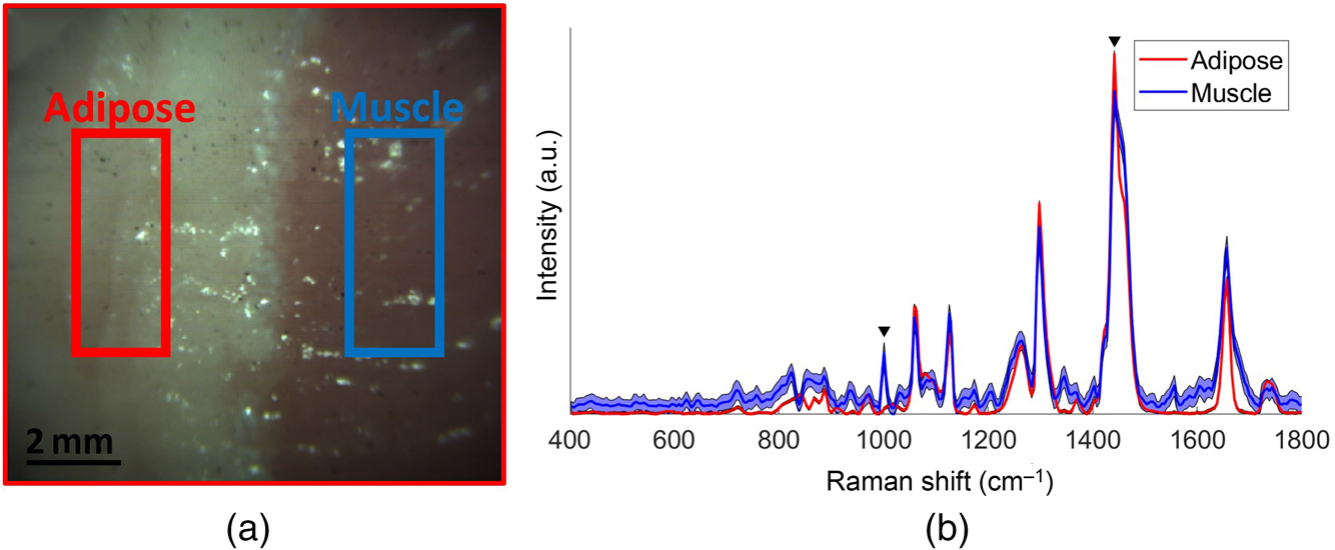
(a) Bright-field image of the sample tissue margin and (b) corresponding mean and standard deviation (shading) of 126 Raman spectra for pork adipose tissue (red) and muscle (blue). Rectangle area corresponds to the selected areas from which the spectra were selected. Black triangles correspond to the two most important features for tissue discrimination identified by the L1-SVM feature selection tool.

A Raman image was then acquired on a tissue margin between adipose and muscle tissue [Fig. 6(a)]. The corresponding mean spectra and standard deviation for regions in both tissue types were extracted [Fig. 6(b)]. This measurement revealed that spectra in muscle tissue were not entirely consistent with the training data set introduced in Sec. 3.2. Pearson coefficients were calculated to evaluate the correlation between the average spectra of pure tissue types in the training set in Fig. 5(c) with those of adipose and muscle tissue of the margin in Fig. 6(b). The Pearson coefficients for adipose tissue within the margin compared with pure muscle and pure adipose tissue were 0.776 and 0.995, respectively, whereas those for muscle within the margin compared with pure muscle and pure adipose tissue were 0.889 and 0.955, respectively. This indicates that the muscle spectra within margins correlate more with the spectra of the pure adipose tissue.

**Fig. 7:**
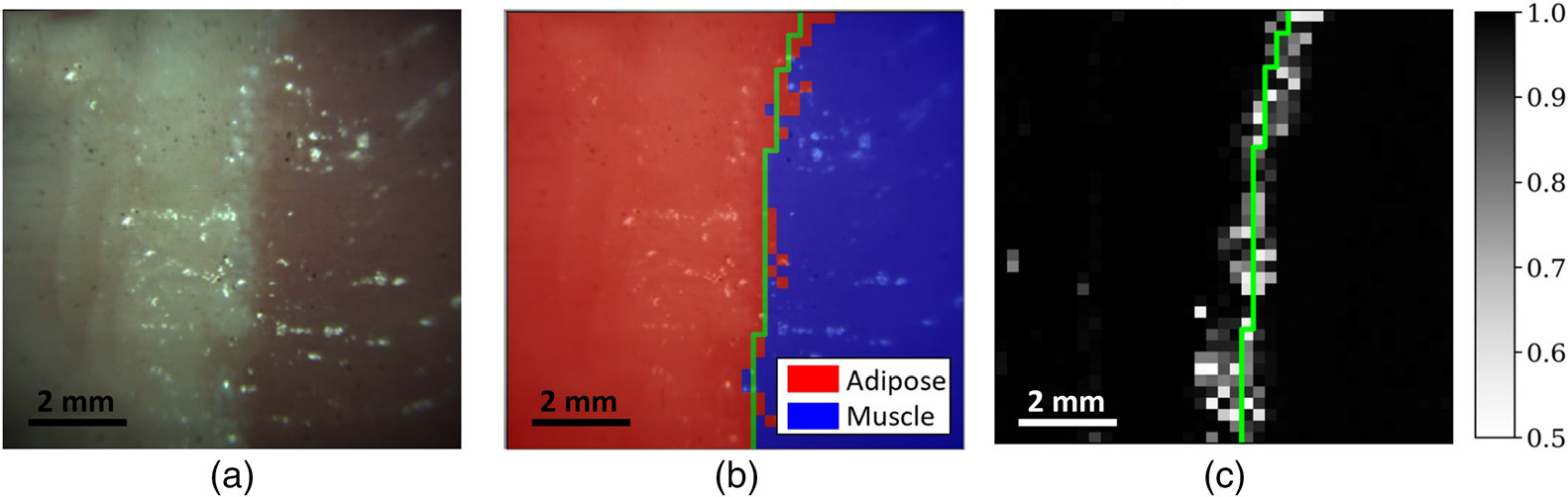
(a) Bright-field image of the sample tissue margin; (b) classification map with red indicating predicted adipose tissue and blue indicating predicted muscle tissue; and (c) classification confidence map ranking from 0.5 (lowest confidence) to 1.0 (highest confidence). The green line shows the ground truth for the tissue margin location.

Following the training dataset standardization, the feature selection step retained only three non-zero-weighted features (i.e., Raman bands): 1001, 1437, and $1442\;{\mathrm{cm}}^{-1}$. These features were then used to train the classification models. The optimized SVM and RF models achieved accuracy scores of 0.990 and 0.967 on the validation set, respectively. Because it had the highest validation accuracy score, the SVM, with hyperparameters C=0.1 and γ=10, was chosen as the model to generate and illustrate the prediction results and their respective class probabilities over the tissue margin in Fig. 7. The accuracy, sensitivity, and specificity of the classification model to discriminate adipose tissue from muscle within the margin are 0.980, 0.996, and 0.958, respectively.

## 4 Discussion

### 4.1 Optical Design

An apparent weakness of the system consists in the resulting line profile at the face of the EFB, for which the laser line width (160  μm) is 60% larger than what was targeted (100  μm). The optics for the excitation branch were designed to accommodate a multimode fiber input of 0.22 NA and 400-μm core. However, the only available laser at the time had a 0.5-NA and a multimode core of 400  μm, which resulted in a larger line width spread in the laser line profile. The impact of this line spread is the generation of a laser line with a width of 400  μm at the sample (after a magnification of 2.5 by the imaging probe optics) as opposed to a targeted width at the sample of 250  μm. A larger laser line produces more in-depth signal from neighboring tissue, which translates into unwanted noise. Additionally, a total of 87% of the laser power is lost through the optics of the excitation branch and the imaging probe due to this difference in fiber specifications. These limitations are expected to be rectified with the integration of a 785-nm laser designed to relay 5 W of power through a 0.22-NA multimode fiber of 400  μm.

A further limitation of the system is the use of fiber bundles for both excitation and collection. Although fiber bundles offer the much-needed range of motion of an imaging probe designed for a clinical context, they present significant optical loses (55% to 65%): first, through the limited effective collection area of the several thousand fibers within the fiber bundles and, second, through the propagation losses within the bundle itself, which are dependent on bundle length. However, the ability to generate the laser line scanning at the excitation branch through fiber bundles offers the advantage of not having any electrical or moving components in the imaging probe, therefore, significantly reducing risks to the patient and allowing for a more robust probe design. Flexibility versus optical loses is a trade-off deemed necessary for user practicality in a surgical context and, therefore, requires significant efforts to mitigate additional optical losses.

Off-the-shelf lenses were selected when available, and two custom lenses were manufactured for the imaging probe design. The aluminum probe casing was fabricated to accommodate the selected optics. The resulting probe size and weight of 0.6 kg could be considered cumbersome for some clinical applications, especially if the probe is to be handheld. Lens size customization will contribute to reducing the probe body’s footprint while a plastic mold approach may reduce overall probe dimensions constrained by machining tolerances and component assembly that are required by a metal casing. The mounting of the probe on a custom apparatus, which is itself mounted on a surgical table (e.g., with a Mayfield^®^ skull clamp), could provide a suitable alternative to a lighter handheld probe.

The resolution criterion through the OTF for illumination and collection across the different parts of the system was easily met across the entire circumradius of the different components with the exception of bright-field imaging spatial resolution within the imaging probe in which the criterion was not quite met in the corners of the FOV. However, the reduction of spatial resolution in the corners of the FOV in bright-field imaging does not hinder the ability to visualize tissue structures.

### 4.2 Raman Imaging of Biological Samples

Raman spectra in both adipose tissue and muscle tissue were collected and totaled 5040 spectra. In the averaged spectra recovered from adipose and muscle tissue, 16 prominent Raman bands along with their biological relevance were identified. The spectra and selected bands showed strong correspondence to previous works in the literature,^37^^,^^45^^–^^47^ in which several Raman bands (620, 824, 853, 1001, and $1604\;{\mathrm{cm}}^{-1}$) are prominent and are associated with the high protein content of muscle, whereas others (967, 1082, 1298, 1346, and $1739\;{\mathrm{cm}}^{-1}$) are associated with the high lipid content of adipose tissue. Many Raman bands (886, 1063, 1126, 1264, 1442, and $1657\;{\mathrm{cm}}^{-1}$) are prominent in both tissue types with varying relative intensities. Sixteen prominent Raman bands were identified although the SNR for each averaged spectrum allowed for the identification and interpretation of many more Raman bands. These other Raman bands were not presented in this work since its aim was to present the imaging system’s ability to acquire high SNR Raman spectra within tissue and use few Raman features for tissue discrimination within an image. Within the entire FOV, spectra were acquired with negligible variance in the highly scattering adipose tissue, whereas muscle tissue spectra showed slightly more variance, which is indicative of a difference of overall SNR for identical acquisition parameters. Comparing adipose and muscle tissue spectra, we observe several distinct high SNR Raman bands, such as the phenylalanine band at $1001\;{\mathrm{cm}}^{-1}$, which was only present in muscle tissue, and the $1442\;{\mathrm{cm}}^{-1}$ band with a higher relative intensity in adipose tissue. These molecular contrasts expressed in the Raman spectra suggest that a low-complexity classifier should successfully discriminate these tissue types.

### 4.3 Margin Identification

Although spectra were significantly distinct when line scanning pure tissue types, this was not the case when the imaged tissue contained margins, i.e., several tissue types within one image. The mean spectra of adipose and muscle tissue interrogated within a tissue margin both correlated much stronger with spectra acquired in pure adipose images. This can be attributed to the orientation of the line measurement in accordance with the tissue margin (in this case perpendicular to the margin), which introduces an in-depth contribution of signal due to the penetration depth of scattered light within tissue (laser and tissue luminescence) and the optical properties of these tissue.^48^ The line profile of the laser generates an in-depth signal within the sample that is ultimately collected across the detection line of the system. This is a phenomenon exploited in spatially offset Raman spectroscopy (SORS) when specific in-depth interrogation is the goal, but in this study, it is an undesirable confounding factor. One notable contribution to an unwanted in-depth signal originates from using a wider laser line profile (400  μm) than that of the collection line (104  μm). The larger the mismatch between the illumination and collection area is, the larger the contribution from in-depth tissue to the overall Raman signal is. It was reported that adipose tissue possesses a very strong Raman cross section ^49^ and is a higher scattering tissue than muscle for the range of 810 to 922 nm,^50^ which would explain the high SNR observed in adipose tissue and its significant spatially offset signal recovered across the detection line in the margin image. If line scanning was performed parallel to the margin presented in Fig. 6, such cross contamination of signal may not have been so apparent. Even so, it was shown that validation results for pure tissue and tissue margin, i.e., accuracy, specificity, and sensitivity, all exceeded values of 97%, with only three selected Raman bands identified to achieve these results: 1001 (phenylalanine breathing mode), 1437 (${\mathrm{CH}}_{2}$ symmetric deformation), and $1442\;{\mathrm{cm}}^{-1}$ (${\mathrm{CH}}_{2}/{\mathrm{CH}}_{3}$ antisymmetric deformation). Although only three bands were selected by the feature selector, this does not exclude the biological relevance and the pertinence of other Raman bands identified in Sec. 4.2 for adipose and muscle tissue discrimination. It does, however, signify that, from the data available, we can generate a very simple yet effective classifier using only these three Raman features, although it is likely that including more spectral features would help in providing accurate tissue class predictions in spectra with an inferior SNR. This classification model could further be simplified by acknowledging that 1437 and $1442\;{\mathrm{cm}}^{-1}$ are both indicative of the same biological content of tissue. Considering the prevalence of phenylalanine in muscle in opposition to its absence in adipose tissue and the large cross section of the long hydrocarbon chains in adipose tissue, rich in ${\mathrm{CH}}_{2}$ symmetric deformation and ${\mathrm{CH}}_{2}/{\mathrm{CH}}_{3}$ antisymmetric deformation, these extracted Raman bands are biologically relevant for tissue discrimination using molecular imaging. The consideration of these tissue specific bands and successful classification validation shows that, in a worst-case scenario, i.e., significant contribution of signal from neighboring tissue, a robust classifier can still successfully discriminate tissue types in macroscopic line scanning with very few Raman bands.

The implementation of a class probabilities map in Fig. 7(c) visually and clearly presents the location of the tissue margin. The biological relevance of this class probability map is that in measurement conditions in which tissues can be discriminated effectively with a classifier, there is a diffuse area corresponding to the margin between tissue types for which a well-trained classifier will gradually have more difficulty in making a prediction from one tissue to the next, due in part to a contribution of Raman scattering that is gradually occurring from a new class of tissue. As the predicted pixel gets closer to the margin, the prediction probability for that class will drop toward its minimum of 50% until the predicted class changes to the new tissue type, gradually increasing as the measurement is further from the margin and as the signal occurs only from the other tissue type. In fact, we observe that, in both tissue types, the classifier confidence is overall near perfect when moving away from the margin. It is acknowledged that visual identification of the margin location based on tissue color and texture does not take into account the thickness or the heterogeneity of the tissue margin (i.e., mixture of adipose and muscle tissue) in the imaged sample and only allows for an approximation of the margin location. Histological analysis of the sample would provide the precise margin size and cellular content crucial for clinical applications, but it was not considered relevant for the conclusions presented in this work. It is estimated visually that the margin presented in Fig. 7 is <1  mm in width, which corresponds to <4  pixels in the image. This is consistent with the probabilities map of Fig. 7(c), which highlights the margin location with an average width of 4 pixels having a prediction confidence of <90%.

Finally, this classification example was purposefully tested with tissue classes trivial to discriminate, adipose versus muscle tissue, to illustrate the functionality of the system and an example of margin recognition capability in only one sample. Using the same sample to interrogate only one area of adipose and muscle tissue, as well as a tissue margin, presents limitations in creating a machine learning classifier that generalizes well to other samples. In this study, measurements resulted in relatively low spectra variance within tissue types and across all Raman images. This led to the creation of classifiers with high validation scores (i.e., SVM and RF accuracy of 99.0% and 96.7%, respectively) indicating that only three bands (i.e., 1001, 1437, and $1442\;{\mathrm{cm}}^{-1}$) can be used to discriminate adipose and muscle tissue. Raman spectra of porcine tissue from previous publications also present the absence of $1001\;{\mathrm{cm}}^{-1}$ phenylalanine band in adipose tissue,^37^^,^^46^^,^^47^ suggesting that our selected features and model could be generalizable to other samples. This will be confirmed in a future study following system improvements. In tissues that present more subtle differences in Raman SNR and band locations such as in diffuse glioma, more imaged samples for training and testing are necessary to capture intersample and intersubject variability.

### 4.4 Photonic Budget Limitation of the System

The results of this study have been observed in a novel line scanning system that was initially designed to obtain comparable signal levels to a single-point probe system employed in surgical guidance for glioma surgery.^39^ Although preliminary data to identify tissue margins is promising, it was observed that the photonic budget (total Raman photons collected) of the line scanner is likely insufficient to generate spectra with sufficiently high SNR spectra for margin identification in tissue with more subtle molecular difference such as brain in an adequate timeframe (<10  min). Table 3 illustrates the key differences in photonic budget for an equal interrogation surface between the line scanner and the above-mentioned point system to explain the performance differences. It is apparent that, in current conditions, the photonic budget collection of the line-scanner when compared with that of the point probe is ∼9600% inferior for the same interrogated unit area although the line scanner acquires 500% greater unit area per line. Although fiber bundle-related losses and the number of collecting fibers (where collecting fibers refer to the collection geometry of the point probe in which multiple fibers participate in the collection of the same detection area) are inherent to these two systems, it is estimated that by implementing a more powerful fiber-coupled 785-nm laser (5 W) with a NA of 0.22 matching the NA of the excitation branch we will decrease power losses and increase the total laser intensity at the sample by >600%. By additionally customizing the spectrometer to obtain an NA that matches that of the collection branch, it is expected that these modifications will result in a realistic increase of >1200% in the collected Raman signal for the same acquisition time and interrogated unit area. A high-power laser (5 W at 785 nm) may cause concern for a clinical setting; however, what is most important is that laser exposure to tissue never exceeds the maximum permissible exposure for skin set by the American National Standards Institute Z136 laser safety standards. A higher laser intensity offers the opportunity to quickly scan the target surface multiple times, leaving ample time between scans for tissue to cool down and thus avoid thermal damage. Further modifications including increasing the NA of the system to 0.36 (f#=1.4) by means of a larger non-contact probe with NIR collection independent of fiber bundles may reduce the photon budget difference between the single-point system and the line scanner to only 50%. With an added change in detection line dimensions, from 104  μm×9.5  mm to 400  μm×10  mm, such a line scanner would interrogate the equivalent area of 20 point-probes for every line.

**Table 3:** Parameter differences between Raman line scanning and Raman point systems comparing the overall photon collection budget for an identical interrogated area and acquisition time.

Parameters	Point system	Line scanning system	Ratio point/line
Laser intensity on sample	$38.2\;W/{\mathrm{cm}}^{2}$	$6.1\;W/{\mathrm{cm}}^{2}$	6.3
Fiber bundle transmission	N/A	37.5%	2.67
Collection branch NA	0.22	0.159	1.96
Number of collecting fibers	7×41.7%^a^	1	2.92
Photon budget	—	—	96

a The point probe contains seven collection fibers that have an effective collection area of 41.7%.

## 5 Conclusion

This paper reports the development of a label-free, large FOV line scanning Raman system, created to address the clinical need of molecular image-guided surgery. The layout of the system and its optical design are detailed, and a proof of functionality on porcine tissue is presented. The system can acquire 1680 high SNR Raman spectra in the fingerprint region over an FOV nearing $1\;{\mathrm{cm}}^{2}$ in <3  min in tissue. With data from pure porcine adipose and muscle tissue, SVM and RF classifiers were trained and validated on a tissue margin with a >97% accuracy, showing the potential of this molecular imaging system to locate tissue margins based on machine learning approaches. Feature selection led to the generation of simplified classification models using only three Raman bands to discriminate porcine adipose from muscle tissue. A detailed discussion addressed the necessary modifications to take this system to a clinical study in human patients to evaluate its potential in locating disease margins *in vivo*.
